# New Drugs, Appliances, and Things Medical

**Published:** 1891-04-04

**Authors:** 


### NEW DRUGS, APPLIANCES, AND THINGS MEDICAL.

[All preparations, appliances, novelties, etc., of which a notice is desired, should be sent for The Editor, to care of The Manager, 140, Strand, London, W.C.]

### ANTISEPTIC DRESSINGS, &c.

Mr. John Milne, Ladywell Antiseptic Dressings Factory, S.E., has forwarded to us a sample case of the above for notice. Coming as they do from the original maker of such goods for Sir Joseph Lister, we should expect the best forms of dressings for maintaining asepsis after and during operations, and we are not disappointed. We can add our emphatic testimony to the value of these dressings, having used them for some years past with comfort to ourselves and safety to our patients. First, then, we have the various forms of sal alembroth preparations, wool, capable of absorbing fifteen times its weight of water, lint and absorbent bandages. Next, Sir J. Lister's last form of aseptic gauze, the Double Cyanide, which, we are informed, is used in nearly every one of the leading hospitals, not only in the United Kingdom, but also very largely abroad as well. Some surgeons prefer iodoform to mercurial dressings, so Mr. Milne has provided for them with excellent applications, gauze (20 per cent.), wool and lint (10 per cent.); and for those who like tow as a dressing, there is the Red Cross antiseptic, carbolised tow (10 per cent. carbolic acid crystals and 25 per cent. pine tar). Amongst the contents of the case we were pleased to note an open-wove bandage for plaster-of-Paris, the particular value of which lies in the fact that it is two yards longer than the usual run of such bandages. No one who has made these bandages will fail to appreciate the value of these extra two yards, saving, as they often will, the making of an extra bandage, of which only a little may be used, the remainder being wasted or being coiled on the limb, uselessly adding to the weight of the splint.

Then we have long glass flasks with catgut ligatures, so arranged that they can be drawn out at once without having to unwind them, and ready cut to a proper length, so that as fast as they are wanted they can be extracted and used. The aseptic catgut is kept in highly carbolised oil. Then there are silk ligatures on glass reels, both Alembroth prepared and carbolised. These can be placed in the trays and the lengths cut off as required. Added to these are the flasks with drainage tubes in antiseptic fluids. Mr. Milne has a useful stand for hospital operating theatres, in which these appliances are so arranged on a large scale that the surgeon can at once make use of any sized ligature, suture, or tube that he may require.

The dressings are put up in large or small packets. Personally, we always order the small packets, as then only as much of a material as is wanted for a dressing is used, the essence of the using being in the statement, "Don't open the packet till the dressing is applied," and thus no soiling of remainder occurs, and the next patient gets an absolutely antiseptic dressing, and not one which possibly has become contaminated. When we are constantly seeing the pink packets of Milne's productions in use at hospitals and brought out of the cases of surgeons at private operations, there is little left for us to say in praise of them. Suffice it to say we know of none better.

